# Hospital sink traps as a potential source of the emerging multidrug-resistant pathogen *Cupriavidus pauculus*: characterization and draft genome sequence of strain MF1

**DOI:** 10.1099/jmm.0.001501

**Published:** 2022-02-03

**Authors:** James Butler, Sean D. Kelly, Katie J. Muddiman, Alexandros Besinis, Mathew Upton

**Affiliations:** ^1^​ School of Engineering, Computing and Mathematics, Faculty of Science and Engineering, University of Plymouth, Plymouth PL4 8AA, UK; ^2^​ School of Biomedical Sciences, Faculty of Health, University of Plymouth, Plymouth PL4 8AA, UK; ^3^​ Peninsula Dental School, Faculty of Health, University of Plymouth, Plymouth PL4 8AA, UK

**Keywords:** Sink trap, *Cupriavidus pauculus*, MF1, whole genome sequencing, *Galleria mellonella*, biofilm

## Abstract

**Introduction.** *Cupriavidus pauculus* is historically found in soil and water but has more recently been reported to cause human infection and death. Hospital sink traps can serve as a niche for bacterial persistence and a platform for horizontal gene transfer, with evidence of dissemination of pathogens in hospital plumbing systems driving nosocomial infection.

**Gap Statement.** This paper presents the first *C. pauculus* strain isolated from a hospital sink trap. There are only six genome assemblies available on NCBI for *C. pauculus *; two of these are PacBio/Illumina hybrids. This paper presents the first ONT/Illumina hybrid assembly, with five contigs. The other assemblies available consist of 37, 38, 111 and 227 contigs. This paper also presents data on biofilm formation and lethal dose in *Galleria mellonella*; there is little published information describing these aspects of virulence.

**Aim.** The aims were to identify the isolate found in a hospital sink trap, characterize its genome, and assess whether it could pose a risk to human health.

**Methodology.** The genome was sequenced, and a hybrid assembly of short and long reads produced. Antimicrobial susceptibility was determined by the broth microdilution method. Virulence was assessed by measuring *in vitro* biofilm formation compared to *Pseudomonas aeruginosa* and *in vivo* lethality in *Galleria mellonella* larvae.

**Results.** The isolate was confirmed to be a strain of *C. pauculus *, with a 6.8 Mb genome consisting of 6468 coding sequences and an overall G+C content of 63.9 mol%. The genome was found to contain 12 antibiotic resistance genes, 8 virulence factor genes and 33 metal resistance genes. The isolate can be categorized as resistant to meropenem, amoxicillin, amikacin, gentamicin and colistin, but susceptible to cefotaxime, cefepime, imipenem and ciprofloxacin. Clear biofilm formation was seen in all conditions over 72 h and exceeded that of *P. aeruginosa* when measured at 37 °C in R2A broth. Lethality in *G. mellonella* larvae over 48 h was relatively low.

**Conclusion.** The appearance of a multidrug-resistant strain of *C. pauculus* in a known pathogen reservoir within a clinical setting should be considered concerning. Further work should be completed to compare biofilm formation and *in vivo* virulence between clinical and environmental strains, to determine how easily environmental strains may establish human infection. Infection control teams and clinicians should be aware of the emerging nature of this pathogen and further work is needed to minimize the impact of contaminated hospital plumbing systems on patient outcomes.

## Introduction

The genus *Cupriavidus* is a member of the family *Burkholderiaceae* and consists of Gram-negative peritrichously flagellated bacilli. *Cupriavidus* species are primarily environmental organisms found in soil [1–3] but have also been isolated from a wastewater treatment plant [4], a groundwater remediation system [5], a hydrotherapy pool [6], bottled mineral water [7], nebulization solutions [8], and even the International Space Station [9, 10]. Many historical isolates were first identified as ‘CDC Group IVc-2’, which was later classified as a *Ralstonia* species [11], then as *Wautersia* [12], before finally being transferred to the genus *Cupriavidus* [13]. Certain *Cupriavidus* species such as *Cupriavidus pauculus* are emerging as opportunistic pathogens of interest [14] with multiple case reports of infection in immunocompromised [15–19] and immunocompetent [20–23] people as well as in those with cystic fibrosis [24–26], with evidence of nosocomial transmission [27–29]. A comprehensive summary of bacteraemia caused by *Cupriavidus* species reported that *C. pauculus* accounted for the vast majority (87 %) of 23 reported cases across different countries [28].

The susceptibility of epidemiologically unrelated *Cupriavidus* spp. clinical strains to 20 antibiotics has been characterized [30], revealing widespread resistance to amoxicillin, amoxicillin-clavulanate, temocillin and aztreonam, relatively low (23 %) susceptibility to ceftazidime, and higher susceptibility to ceftriaxone and cefotaxime (74 and 82%, respectively). Mixed susceptibility to colistin was reported, which is significant as the *Cupriavidus* genus was initially described as being colistin-susceptible [31] but *C. metallidurans* was later found to exhibit high expression of aminoarabinose transferase (ArnT) [32], a colistin resistance determinant, and *C. gilardii* may have been the origin of the *mcr-5* gene [30, 33].

Sink traps (also known as U-bends or P-traps) are sections of wastewater plumbing systems (WPS), both domestic and commercial, designed to trap water and prevent unwanted flow of sewer gases into the sink and surrounding environment. Sinks are present in virtually all hospital wards and patient rooms to encourage best practice regarding hand hygiene [34, 35], however these sink traps become heavily colonized with pathogenic bacteria and are an important reservoir of pathogens causing nosocomial infections [36–40]. The formation of biofilms, which are refractory to disinfection and facilitate long-term persistence of bacteria, poses an additional concern [41]. As well as acting as a reservoir, sinks are points of direct pathogen dispersal when they are used as bacteria escape by droplets and aerosols [42–45], and bacteria can also be carried on airflows within plumbing systems even between different floors of a building [46]. Furthermore, due to the realistic nature of sinks being used as an occasional point of disposal of patient material, pharmaceuticals and/or disinfectants, their resident bacteria are exposed to a wide variety of chemical substances. One study of an intensive care unit found that only 4 % of total sink-related behaviours could be categorized as handwashing, and a total of 56 sink-related activities could be identified that introduced a variety of nutrients potentially promoting microbial growth in the sinks [47]. There is evidence to suggest that hospital effluent wastewater selects for antibiotic resistance [48], suggesting that surfaces in the WPS may become enriched with resistant bacteria and so WPS could be further thought of as a reservoir of resistance genes. These resistance genes can reside in various genera of hardy environmental organisms and sinks may provide a platform for horizontal gene transfer [49]. *C. pauculus* has previously been linked with clinical WPS: in one case a *C. pauculus* bacteraemia was linked to a contaminated hospital water system [50] while in another case a ‘pseudo-outbreak’ at an outpatient clinic was linked to rinsing culture swabs in tap water [51], however isolation of *C. pauculus* from a hospital sink trap has not previously been reported.

In this article, we report the annotated draft genome sequence of *C. pauculus* strain MF1 isolated from a sink trap in a UK hospital. We also report the isolate’s genotypic and phenotypic antimicrobial susceptibility profile, determination of median lethal dose in *Galleria mellonella* larvae, and biofilm formation compared to *Pseudomonas aeruginosa *.

## Methods

### Sample collection and bacterial recovery

Sink traps (*n*=2) were removed from sinks at a UK hospital in 2017 by hospital staff, sealed in individual sample bags, transferred to University of Plymouth laboratories, and stored at −20°C. In 2020, sink traps were thawed to room temperature (RT) for 1 h before sampling with a sterile swab moistened with Dulbecco A PBS (Thermo Scientific Oxoid, UK). Swabs were placed in tubes containing 3 ml PBS, then rotated and vortexed. Suspensions were serially diluted tenfold to 10^−3^ and all subsequent suspensions were spread plated in 100 µl aliquots on columbia blood agar (CBA) composed of columbia agar (Sigma-Aldrich, MO, USA) with 5 % (*v/v*) defibrinated horse blood (Fisher Scientific, UK). All bacteriological media and PBS were sterilized by autoclaving at 121 °C and 15 psi for 15 min. Agar plates were incubated at either 20 °C or 37 °C for up to 7 days.

### Gram stain

To visualize cell morphology, indicate cell-wall composition and ultimately aid in initial identification of the organism, a Gram stain was conducted. A few colonies of the isolate were emulsified in a small volume of sterile deionized water spread across a glass slide, allowed to dry to give a smear, and fixed by briefly passing through a Bunsen flame. The slide was stained for 1 min by flooding with crystal violet (CV) solution (Pro-Lab Diagnostics, UK), rinsed with tap water, then flooded with the mordant Gram’s iodine (Pro-Lab Diagnostics, UK) for 1 min before rinsing briefly again with tap water. The slide was then held on a slant while a few drops of the decolourizing agent 95 % ethanol were added and rinsed off with tap water. Finally, the counterstain safranin (Pro-Lab Diagnostics, UK) was added for 1 min and rinsed off with tap water. The slide was blotted dry and visualized with a brightfield microscope.

### Strain identification by 16S rRNA gene sequencing

Identification was continued based on the 16S rRNA gene sequence. Genomic DNA was extracted from a pure culture using the DNeasy PowerSoil Kit (Qiagen, Germany), according to the manufacturer’s instructions. This kit utilizes an initial bead-beating step to lyse cells, followed by binding to a silica membrane within a spin column in the presence of chaotrophic salts, removal of contaminants, and finally elution of purified DNA from the membrane. The 16S rRNA gene was amplified by PCR in 50 µl reactions consisting of 25 µl DreamTaq Green 2× PCR Master Mix (Thermo Fisher Scientific, UK), 5 µl DNA template, 2.5 µl of each primer (Eurofins Genomics, Luxembourg) at 0.5 µM (27 f: 5′–AGA GTT TGA TCA TGG CTC A–3′, 1492 r: 5′–TAC GGT TAC CTT GTT ACG ACT T–3′), and 15 µl Ambion️ nuclease-free water (Invitrogen, MA, USA). A positive control of a known bacterial DNA sample and a negative control of Ambion️ nuclease-free water were included in the place of the DNA template. The thermocycler programme was as follows: 5 min at 94 °C followed by 30 cycles of 30 s at 94 °C, 1 min at 52 °C, and 1.5 min at 72 °C, followed by 10 min at 72 °C. The presence and size of PCR products were confirmed by gel electrophoresis, running 5 µl of each PCR reaction mixture on a 1.5 % agarose gel with SYBR Safe DNA Gel Stain (Invitrogen, MA, USA). PCR products were prepared for sequencing using the ExoSAP-IT PCR Product Cleanup Reagent (Thermo Fisher Scientific, UK) according to the manufacturer’s instructions. Amplicon sequencing was completed by Biosearch Technologies (LGC Genomics, Germany), and sequences were analysed using Geneious Prime 2021.1.1 for macOS (https://geneious.com). The ends of the forward and reverse sequences were trimmed based on quality (error probability limit 0.01) and the sequences aligned using the muscle algorithm (default settings) within Geneious Prime. The resulting consensus sequence was then used for a search using the Basic Local Alignment Search Tool (blast; https://blast.ncbi.nlm.nih.gov/Blast.cgi).

### Antimicrobial susceptibility testing

The guidelines provided by the European Committee on Antimicrobial Susceptibility Testing (EUCAST; https://www.eucast.org) were followed for completion of MIC assays by the broth microdilution method. All MIC assays were conducted in cation-adjusted Mueller–Hinton II broth (Sigma-Aldrich, MO, USA) in 96-well round-bottom polypropylene microplates (Corning, NY, USA) and results determined visually following 18±2 h static incubation at 37 °C. Antibiotics were purchased from Melford Laboratories (amikacin, amoxicillin, amoxicillin-clavulanate, cefotaxime, colistin), Sigma-Aldrich (ceftazidime, ciprofloxacin, doxycycline, erythromycin, meropenem), Carbosynth (imipenem, relebactam), Fisher Scientific (cefepime) and Lonza (gentamicin). Antibiotic stocks were prepared in sterile deionized water, except erythromycin, which was prepared in dimethyl sulfoxide, and stored at −20 °C until use. A 16 : 1 ratio of amoxicillin to relebactam was used, as previously reported [52]. Classification of the isolate’s susceptibility to antibiotics was determined based on EUCAST breakpoints [53] using entries for *Pseudomonas* spp. as published previously [28, 30, 54], as well as PK-PD breakpoints which are not species-specific.

### Draft genome sequence determination

Illumina next-generation sequencing was provided by MicrobesNG (https://microbesng.com). The isolate was submitted in the form of cells harvested from an agar plate after streaking a single colony, suspended in a tube with cryopreservative (Microbank, Pro-Lab Diagnostics, UK). DNA extraction was completed by MicrobesNG using lysozyme, RNase A, proteinase K, and sodium dodecyl sulphate, and genomic DNA was purified using SPRI beads. Details of the DNA extraction, quantification and sequencing library preparation are available at https://microbesng.com/microbesng-faq/. Finally, reads were adapter-trimmed using Trimmomatic 0.30 with a sliding window quality cut-off of Q15.

Oxford Nanopore Technologies (ONT) MinION sequencing was employed to produce long reads to assist in genome assembly. Genomic DNA was isolated using the DNeasy Blood and Tissue Kit (Qiagen, Germany) with a modification made for Gram-negative bacteria according to the manufacturer’s instructions; brief suspension in a lysis buffer (kit buffer ATL). Precautions were taken throughout to reduce shearing of high molecular weight DNA, including flicking tubes to mix rather than vortexing, use of wide bore pipette tips, avoidance of freeze–thaw cycles, and keeping tubes on ice. Extraction yield was quantified using the Qubit dsDNA HS Assay Kit (Invitrogen, MA, USA) and fluorometer (Thermo Fisher Scientific, UK), and purity was determined using a Nanodrop spectrophotometer (Thermo Fisher Scientific, UK). ONT sequencing was completed using the 1D Native barcoding genomic DNA protocol (SQK-LSK108) and EXP-NBD103 barcoding kit on an R9.4.1 flow cell (FLO-MIN106) according to the manufacturer’s instructions. Basecalling was completed using Guppy version 5.0.11 with default settings using the Cloud Infrastructure for Big Data Microbial Bioinformatics (CLIMB) platform [55].

### Hybrid genome assembly and annotation

A hybrid assembled and annotated genome consisting of Illumina short reads and ONT long reads was produced using the Comprehensive Genome Analysis tool provided by the PATRIC 3.6.10 Bioinformatics Resource Center [56]. Unicycler [57] was selected as the assembly strategy and annotation was completed using the RAST toolkit (RASTtk) [58].

### *In vivo G. mellonella* infection to determine median lethal dose of *C. pauculus* MF1

Larvae of the greater wax moth *G. mellonella* were purchased from Livefood UK and stored in the dark at 4 °C for up to 7 days without food or water. Larvae were selected to be 21±1 mm in length and healthy, as determined by a uniform cream colour, with no indications of melanization. Larval length can be correlated to weight according to a linear regression, such that a length of 21±1 mm equates to a weight of 208.2±22.2 mg, which is within the optimum range (180–260 mg) for determination of median lethal dose (LD_50_) using this model [59]. To prepare the *C. pauculus* MF1 inoculum, the isolate was grown overnight in 10 ml LB broth (Fisher Scientific, UK) at 37 °C and collected by centrifugation at 2500 ***g*** for 10 min. The pellet was then resuspended in 10 ml PBS to wash the cells, before repeating the centrifugation once more and finally resuspending the pellet in 1 ml PBS. The resulting suspension was tenfold serially diluted and bacterial density was confirmed by viable count assay using the Miles and Misra method [60].

Ten larvae were injected per serial dilution of *C. pauculus* MF1 (10^2^, 10^3^, 10^4^, 10^5^, 10^6^ and 10^7^ c.f.u./10 µl) into the left penultimate pro-leg using a 50 µl Hamilton 750 syringe (Hamilton Company, NV, USA) with a removable 26S gauge needle. A placebo control of sterile PBS was used to account for the physical trauma of injection (*n*=10), along with a ‘no manipulation’ control to account for normal survival (*n*=10). Injected larvae were placed into sterile Petri dishes and incubated at 37 °C in the dark without food or water for 48 h. After 24 and 48 h, the percentage of live larvae in each treatment group was recorded. Larvae were recorded as dead when they met at least two of the following criteria: (i) obvious melanization, (ii) no response to touch, (iii) no correction when rolled on back [59]. For each time point, the LD_50_ was calculated by plotting c.f.u./larva against percentage live larvae and interpolating the curve for 50 %. The mean average of three biological replicates is reported.

### Biofilm formation compared to *P. aeruginosa* PAO1

The CV solubilization assay was used to measure the biomass of formed biofilm in microplates according to the methods published previously [61–63]. *P. aeruginosa* was selected as a comparator organism due to its wide acceptance as a versatile biofilm-forming organism [64–66], and PAO1 was selected due to its ubiquity in research as a well-characterized strain [67]. *C. pauculus* MF1 and *P. aeruginosa* PAO1 were each inoculated in tryptic soy broth (TSB; Sigma-Aldrich, MO, USA) and Reasoner’s 2A (R2A) broth (prepared in-house identically to Oxoid CM0906 but omitting agar, see the Supplementary Material, available in the online version of this article, for composition) and incubated at 37 °C until log phase. Cultures were then adjusted to 1×10^6^ c.f.u. ml^−1^ and 200 µl/well added to flat-bottom 96-well plates (Thermo Scientific Biolite). There were 12 technical replicates per bacteria-media combination. Outer microplate wells were filled with 200 µl sterile water to exclude any edge effects and a negative control of broth alone was included. Plates were incubated statically at 37 °C or 20 °C for 24–72 h. To quantify biomass of biofilm formed, at each time point plates were washed with dH_2_O to remove nonadherent cells, stained by addition of 200 µl/well 0.1 % (*w/v*) CV solution, and incubated at RT for 20 min. CV was removed by pipette and then wells were washed again with dH_2_O to remove unbound CV. Plates were dried at 60 °C for 30 min before cooling and solubilizing CV by addition of 200 µl/well 30 % (*v/v*) acetic acid and incubating at RT on a shaking platform for 30 min. Absorbance was read at 575 nm using a SPECTROstar Omega (BMG Labtech, UK). The mean absorbance of negative controls was subtracted from that of each experimental well to account for background staining. At each time point, plates not being measured were also refreshed by removing liquid from wells and replacing with 200 µl/well appropriate fresh media.

### Statistical analysis

All experiments consist of three independent biological replicates and at least three technical replicates per group, or more where indicated. Statistical analyses were conducted using GraphPad Prism v9.1.1 for macOS (GraphPad Software).

Data were checked for normal distribution using the Shapiro–Wilk test. Two-way ANOVA was conducted on biofilm data, with Šídák’s multiple comparisons test. All statistical analyses used a 95 % confidence limit, so that *P*-values equal to or greater than 0.05 were not considered statistically significant.

## Results

### Initial isolation and identification of the strain

The isolate appeared as grey round easily emulsifiable colonies on CBA, and Gram-negative bacilli were apparent on staining. Sanger sequencing of the 16S rRNA gene PCR product and subsequent processing yielded a 1392 base pair (bp) consensus sequence. A blast search gave resolution to genus-level, but species-level identification could not be completed due to many 100 % identity and 100 % query cover matches to multiple *Cupriavidus* species (* C. pauculus *: MW960228, MW049071, MW049067, AY860236, AB109753; *C. taiwanensis *: EU915716) and multiple sequences identified only to genus level as *Cupriavidus* (GU566329, EU827490, EU596431).

### Draft genome sequencing

Incorporation of both the Illumina short reads and ONT long reads in a hybrid assembly resulted in a 6.8 Mb draft genome of five contigs, with a ‘good’ quality determination by PATRIC and 0 % contamination. The number of coding sequences (CDS) was 6468 – of which 4063 were given functional assignments. PATRIC indicated that the genome was 99.5 % complete. For further details of the hybrid assembly and annotated genome, see Table S1. Summary statistics from the ONT MinION sequencing run can be found in Table S2 and characteristics of an assembly resulting from Illumina reads alone can be found in Table S3.

Multi-locus sequence typing (MLST) using the autoMLST web tool (https://automlst.ziemertlab.com/) gave the closest match as 98.2 % average nucleotide identity (ANI) to *C. pauculus* KF709 (NBRC 110672) deposited as GCA_000974605 [3]. As the ANI cut-off for new prokaryotic species is given as 95 % [68, 69], the isolate was concluded to be part of the existing species *C. pauculus *. See Table S4 for the top 10 MLST results. To confirm the MLST classification, the Genome-to-Genome Distance Calculator provided by the Leibniz Institute DSMZ was used to run a digital DNA–DNA hybridization (DDH) to mimic DDH *in silico* [70]. This tool uses three independent formulae, reporting three DDH values. The results were 73.8, 85.4 and 78.5 % (mean 79.2 %) against the closest match, *C. pauculus* KF709 as above. A DDH value of ≥70 % is recommended as the threshold for definition of members of the same species [71, 72], thus on this basis the isolate was confirmed to be part of the existing species *C. pauculus *. The strain was designated MF1.

The RASTtk within PATRIC was used to identify antibiotic resistance genes (ARGs), virulence factor genes (VFs) and metal resistance genes (MRGs). The full list of the genes of interest within these categories can be found in Table 1. In total, 12 ARGs, 8 VF genes and 33 MRGs were identified.

**Table 1.** Genes of interest found within the *C. pauculus* MF1 draft genome

Gene type	Gene name	Gene product/function
Antibiotic resistance genes	*AmpC*	β-lactamase
	*adeF, adeG*	RND multidrug efflux pump (AdeFGH)
	*arnT*	Aminoarabinose transferase enzyme conferring colistin resistance
	*bla*_OXA_	Class D β-lactamase
	*ceoB*	Cytoplasmic membrane component of the CeoAB-OpcM efflux pump
	*EmrAB-TolC*	Multidrug efflux system
	*H-NS*	Regulates expression of multidrug exporter genes
	*MacA, MacB*	Form an antibiotic efflux complex with TolC active against macrolides
	*MdtABC-TolC*	Multidrug efflux system
	*MexAB-OprM*	Multidrug efflux system
Virulence factors	*argG*	Argininosuccinate synthase; arginine synthesis
	*aroC*	Chorismate synthase; aromatic amino acid synthesis
	*hfq*	RNA chaperone at the post-transcriptional level
	*leuB*	3-isopropylmalate dehydrogenase
	*ppsA*	Phosphoenolpyruvate synthase
	*recA*	DNA repair and maintenance
	*rpoE*	RNA polymerase sigma E; facilitates bacterial growth at high temperature and may facilitate persistence in macrophages
	*trpG*	Anthranilate synthase component
Metal resistance genes	*acr3*	Transmembrane arsenite transporter
	*arsH*	Methylarsenite oxidase; ferric reductase activity and protection from oxidative stress
	*cbtA*	Putative cobalt transporter subunit
	*cnrR, cnrY*	Nickel and cobalt resistance
	*cobS-T, cobN, cobW*	Cobalt chelatases involved in cobalamin synthesis, *cobW* is a putative zinc chaperone
	*copC-D, copZ*	Copper chaperone and sequestration, may also bind silver and cadmium
	*corA, corC*	Na^+^-dependent Mg^2+^ transporter
	*cusA-C, cusR-S*	Copper and silver resistance
	*cutE*	Copper transport
	*czcA-D, czcI, czcN*	Cadmium, zinc, and cobalt efflux
	*merP, merR, merT*	Mercury resistance
	*modA-C*	Components of molybdate uptake operon
	*nikR*	Transcriptional regulator of the nikABCDE operon

Listed are genes of interest found in the *C. pauculus* MF1 isolate whole genome, as annotated and highlighted by the PATRIC Comprehensive Genome Analysis tool using both CARD and PATRIC databases.

### Susceptibility to antibiotics

The MICs of antibiotics against the *C. pauculus* MF1 isolate and the resulting susceptibility classifications can be found in Table 2. As breakpoint tables for this genus are not available, susceptibility classifications were based on breakpoints for *Pseudomonas* spp. as has been published previously [28, 30, 54], as well as PK-PD breakpoint values, which are not species-specific. The isolate showed susceptibility or intermediate susceptibility to the cephalosporins cefotaxime, ceftazidime and cefepime. For carbapenems, resistance to meropenem was clear however the isolate was susceptible to imipenem with little additional benefit conferred by the inclusion of the β-lactamase inhibitor relebactam. The isolate was resistant to amoxicillin but could be sensitized to some degree by the addition of the β-lactamase inhibitors relebactam or clavulanate, which reduced the MIC by over 32-fold and 8-fold, respectively. Low MICs were observed for both ciprofloxacin (a fluoroquinolone) and doxycycline (a tetracycline) while high MICs were found for both gentamicin and amikacin (aminoglycosides). The MIC of erythromycin was above that which could be considered clinically beneficial, however conclusive classification is not possible due to the lack of a published breakpoint. Finally, the isolate can be classified as resistant to colistin.

**Table 2.** MICs of antibiotics against the *C. pauculus* MF1 isolate

Class	Antibiotic	MIC (µg ml^−1^)	Classification (* Pseudomonas* spp.)	Classification (PK-PD)
Cephalosporins	Cefotaxime	1	n/a	Susceptible
	Ceftazidime	8	Intermediate*	Intermediate
	Cefepime	0.25	Intermediate*	Susceptible
Carbapenems	Meropenem	>512	Resistant	Resistant
	Imipenem	0.5	Intermediate*	Susceptible
	Imipenem-Relebactam (2 : 1)	0.25	Susceptible	Susceptible
Penicillins	Amoxicillin	>512	n/a	Resistant
	Amoxicillin-Relebactam (16 : 1)	16	n/a	n/a
	Amoxicillin-Clavulanate (5 : 1)	64	n/a	Resistant
Aminoglycosides	Gentamicin	512	IE	Resistant
	Amikacin	512	Resistant	Resistant
Fluoroquinolones	Ciprofloxacin	0.125	Intermediate*	Susceptible
Macrolides	Erythromycin	32	n/a	IE
Tetracyclines	Doxycycline	0.25	n/a	IE
Polymyxins	Colistin	16	Resistant	IE

MICs of antibiotics against the *C. pauculus* MF1 isolate and the resulting susceptibility/resistance classifications. Classifications according to the breakpoints for *Pseudomonas* spp. are given as well as classifications based on pharmacokinetic-pharmacodynamic data provided by EUCAST. ‘N/A’ indicates that a breakpoint is not available. ‘IE’ indicates that EUCAST report there is insufficient evidence to consider a particular agent as a therapy and give no breakpoint. Intermediate with an asterisk (*) indicates that an MIC breakpoint of S≤0.001 µg ml^−1^ is given as an ‘arbitrary off scale’ breakpoint by EUCAST. Where any set of results contained differences of more than one serial dilution step, the assays were repeated. Shown are the consensus (mode) results of three biological replicates; alternatively, the midpoint was used if the three results were within one serial dilution step of each other.

### Median lethal dose in *G. mellonella* larvae

The 24 h LD_50_ of *C. pauculus* MF1 in *G. mellonella* was determined to be 2.54×10^7^ c.f.u./larva (sd±5.99×10^6^) while the 48 h LD_50_ was determined to be 2.64×10^6^ c.f.u./larva (sd±1.82×10^6^). Taking the calculated average weight of larvae used in this study as 208.2 mg, these LD_50_ values equate to 1.22×10^5^ c.f.u. mg^−1^ (24 h) and 1.27×10^4^ c.f.u. mg^−1^ (48 h).

### Biofilm formation compared to *P. aeruginosa* PAO1

**Fig. 1.**
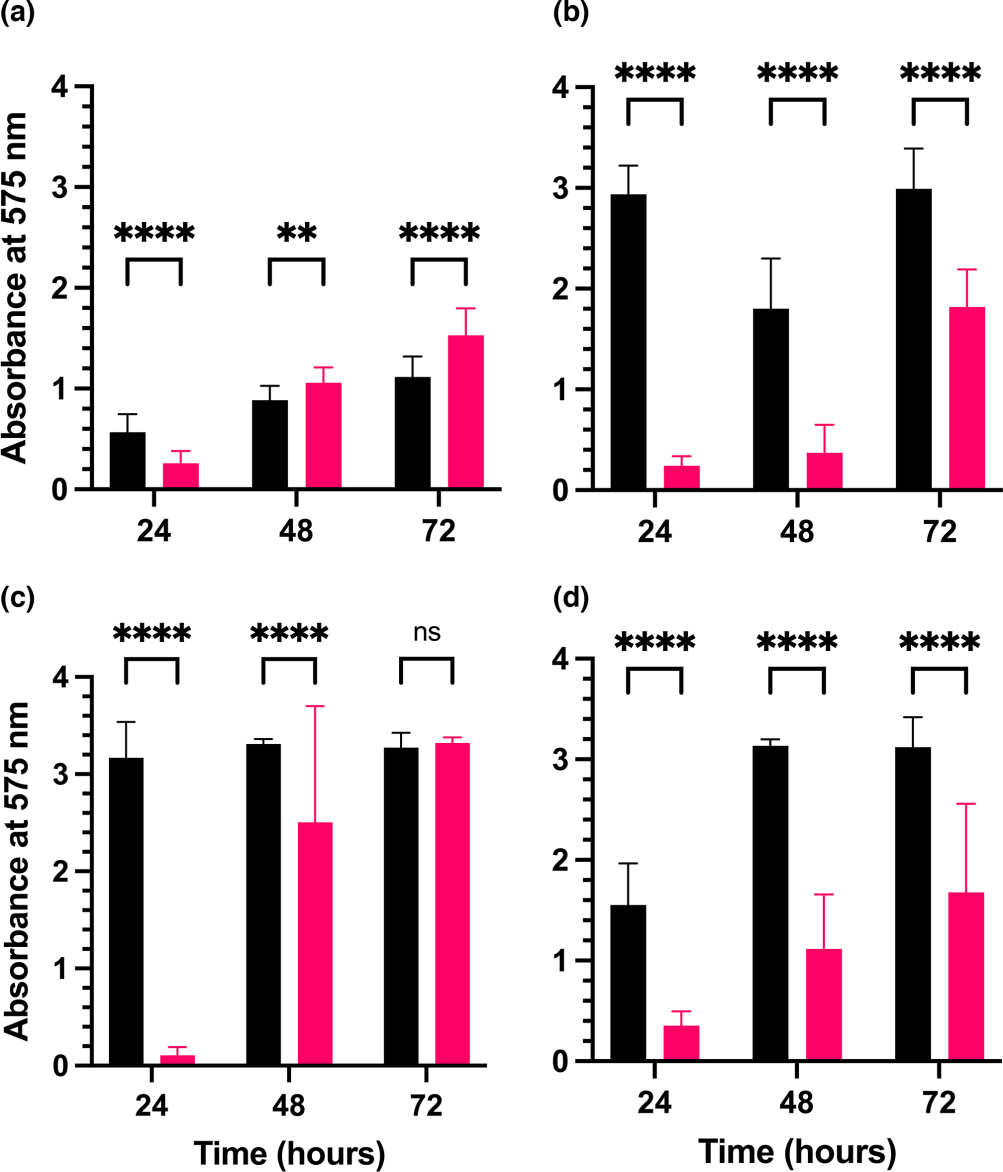
Biofilm formation by *C. pauculus* MF1 (pink) and *P. aeruginosa* PAO1 (black) in R2A broth at either 37 °C (a)or 20 °C (b)and in TSB at either 37 °C (c)or 20 °C (d)over 72 h as determined by the CV solubilization assay. Data presented as mean±sd. **** *P*<0.0001, ** *P*<0.01, ns: not significant; two-way ANOVA with Šídák’s multiple comparisons test. Data presented are the average of *n*=3 biological replicates.

Both *C. pauculus* MF1 and *P. aeruginosa* PAO1 formed detectable biofilms in minimal (R2A) and rich (TSB) broth at both physiological (37 °C) and environmental (20 °C) temperatures between 24–72 h (Fig. 1). In R2A broth at 37 °C, *C. pauculus* biofilm built gradually and significantly overtook that of *P. aeruginosa* after 48 and 72 h (Fig. 1a). In R2A broth at 20 °C, *P. aeruginosa* formed much more biofilm, almost reaching maximum signal after only 24 h, while *C. pauculus* biofilm steadily built across the whole 72 h, though not overtaking that of *P. aeruginosa* under the same conditions (Fig. 1b). Generally, biofilm levels reached in TSB were greater than in R2A broth (Fig. 1c, d). In TSB at 37 °C, the *C. pauculus* biofilm climbed from a low level at 24 h and reached a similar high level as *P. aeruginosa* after 72 h (Fig. 1c). In TSB at 20 °C, *P. aeruginosa* biofilm levels reached similar high levels after 48 h onwards but at 24 h showed clearly less biofilm than at 37 °C (Fig. 1d). The *C. pauculus* biofilm built across 24–72 h in TSB at 20 °C but did not reach the same level as *P. aeruginosa* by the endpoint (Fig. 1d).

## Discussion

*C. pauculus* is an emerging human pathogen, having previously been chiefly isolated from soil and aquatic environments. Here, we present its isolation from a hospital sink trap – a niche known to act as a reservoir of pathogens in clinical settings and contribute to nosocomial transmission [36–40]. This study reports the draft hybrid genome sequence of the isolate and investigates its antimicrobial susceptibility, virulence in an *in vivo* model, and biofilm formation in relevant conditions compared to a well-characterized biofilm-forming model organism.

The isolate could not be identified to species-level using 16S rRNA gene sequencing; WGS was therefore indicated for identification and to further investigate the organism. A hybrid assembly of ONT and Illumina reads allowed a more complete genome to be achieved, adding to only six other genomes available for this species – two of which are PacBio/Illumina hybrids. Of these hybrids, both (GCA_008693385 and GCA_003854935) were submitted as part of the same FDA-ARGOS [73] project (PRJNA231221) in 2019 and consist of two contigs with one and two plasmids, respectively. The number of CDS identified in this study (6468) was greater than in both available hybrid genomes (6286 and 5483) although there was a difference in annotation method (RASTtk in PATRIC for this study vs NCBI Prokaryotic Genome Annotation Pipeline for the FDA-ARGOS project). There was also a difference in total sequence length (6.8 Mb versus 7.1 Mb and 6.2 Mb). Finally, the genome reported in this study did not contain any identifiable plasmids.

Through WGS, several ARGs were identified (Table 1) – mostly components of efflux pumps (*adeG*, *EmrAB-TolC*, *MdtABC-TolC*, *MexAB-OprM*, *MacA/MacB*) as well as other sequences identified by RASTtk as β-lactamases (*AmpC*, *bla*_OXA_). Phenotypic resistance to meropenem, amoxicillin, amikacin, gentamicin and colistin was evident. The β-lactamase inhibitors relebactam and clavulanate reduced the MIC of amoxicillin, suggesting a resistance mechanism involving a β-lactamase, however the resulting MICs were still not within a clinically relevant range. The isolate was clearly susceptible to cefotaxime, cefepime, imipenem and ciprofloxacin. The antibiotic susceptibility profile appeared to broadly match that for *C. pauculus* reported by Massip *et al.* [30], except in the cases of meropenem (>512 μg ml^−1^ here versus 16–64 μg ml^−1^ reported) and amikacin (512 μg ml^−1^ here versus 8–128 μg ml^−1^ reported). Through further analysis of the ARGs identified by RASTtk, it became apparent that certain genes, which only confer resistance following acquisition of point mutations, such as *gyrA* and *rpsJ* [74, 75], are automatically identified as genes of interest – even when they lack the required mutations. Following investigation, these genes were discounted from our reported genes of interest. This highlights the importance of manually checking the output of genome annotation pipelines to confirm the relevance of identified genes.

While none of the mobilized colistin resistance (*mcr*) genes were identified in the genome, the isolate’s phenotypic resistance to colistin may be explained by the presence of an *arnT* homologue with 81 % identity and 99 % query cover against that reported in *C. metallidurans* CH34 (CP000353.2 : 1481129–1482725) [30]. Aminoarabinose transferase (ArnT) catalyses the attachment of the cationic sugar 4-amino-4-deoxy-l-arabinose to lipid A, resulting in charge modification of the bacterial outer membrane and polymyxin resistance [32]. Homologues of *arnT* have previously been described in *C. pauculus *, *C. basilensis *, *C. necator* and *C. taiwanensis* as the cause of colistin resistance [30].

A large number (*n*=33) of MRGs were identified in the WGS. Together, the MRGs listed in Table 1 contribute to resistance to copper, silver, magnesium, cobalt, mercury, molybdenum, arsenic, zinc, cadmium, iron, antimony and nickel. Many species of *Cupriavidus* including *C. pauculus* [76, 77], *C. metallidurans* [78, 79], *C. gilardii* [80], *C. campinensis* [81] and *C. neocaledonicus* [82] are considered to be metal resistant. Many of these genes confer resistance to metals by means of intracellular metal sequestration to facilitate export, as in the case of *CopZ* with Cu^+^ ions [83], Ag^+^ and Cd^2+^ ions [84]. There is a link between metal and antibiotic resistance, in that ARGs can co-locate on mobile genetic elements carrying MRGs such as the integrative conjugative element ICE*Hs*1, which contains 83 genes, including both ARGs (*tetH*, *ebrB*) and MRGs (*mco*, *czcD*, *acr3*) [85].

The RASTtk annotation also identified a number (*n*=8) of VFs. One of these, *hfq*, is a known pleiotropic regulator of virulence genes with the protein Hfq acting as an RNA chaperone and modulating mRNA translation and stability [86]. Another, *recA*, is involved in recombination, repair and maintenance of DNA, as well as induction of prophage [87]. *Salmonella typhimurium* mutants deficient of *recA* have been shown to be less virulent in BALB/c mice and more susceptible to macrophage killing by oxidative burst [88]. The alternative sigma factor *rpoE* is essential for some species such as *E. coli* [89], and is also induced by stressors, including heat and accumulation of misfolded proteins, to trigger extensive alterations in gene expression [90, 91]. RpoE, its product, has been shown to be important for intracellular survival of *Salmonella typhimurium* in macrophages [92]. The *G. mellonella* larvae model, a well-characterized model for virulence and lethality testing, was used to assess median lethal dose of the isolate. Non-lethal isolates (non-pathogenic *E. coli* DH5ɑ) have previously been reported with an LD_50_ of around 10^7^ c.f.u./larva [93], which is around the same range as the 24 h LD_50_ values reported in this study. As would be expected, the 48 h LD_50_ values were lower than those at 24 h but remained relatively high. This suggests that *C. pauculus* MF1 exhibits low levels of virulence, at least in *G. mellonella* in the conditions described. There is some room for interpretation in all findings for *G. mellonella* as authors calculate and report results in myriad ways, e.g. c.f.u./larva versus c.f.u. mg^-1^, and some authors do not report whether they controlled for larval size or weight. Larval weight shows a strong correlation to larval liquid volume [94], and it follows that injections into larger larvae will lead to greater inoculum dilution possibly leading to error in the reported LD_50_. *G. mellonella* are particularly susceptible to killing by *P. aeruginosa *, with a reported LD_50_ of only 10 c.f.u. [95]. Meanwhile the 24 h LD_50_ values of uropathogenic *E. coli* isolates have been determined to be around 1.6×10^4^ c.f.u./larva (ST69) and 1.2×10^4^ c.f.u./larva (ST127) [93]. The 48 h LD_50_ of two strains of *Burkholderia cepacia* have been reported as 30 c.f.u./larva or 1 c.f.u./larva while that of eight strains of *B. cenocepacia* ranged from 9×10^2^ c.f.u./larva to 2×10^5^ c.f.u./larva [96]. It is noteworthy that despite them both belonging to *Burkholderiaceae *, *C. pauculus* MF1 and the strains of *B. cepacia* and *B. cenocepacia* have significantly different LD_50_ values in *G. mellonella*, which may be explained by differences in virulence factors and resulting pathogenicity. The virulence of *Burkholderia* spp. is the result of potent biofilm formation, intrinsic antibiotic resistance, metabolic and genetic plasticity, multiple secretion systems, and the ability to persist intracellularly within macrophages and other eukaryotic cells [97]. Further evaluation of the *in vivo* virulence of this and other *C. pauculus* strains in another model would provide a valuable insight.

Biofilm formation was determined in 96-well plates according to a widely used protocol involving staining and solubilization of CV, which binds to all microbial biomass within each well. The CV solubilization assay therefore quantifies biofilm biomass, but cannot enumerate bacterial cells or indicate their viability. R2A agar was initially developed for enumeration and subculture of bacteria from potable water [98], and so we propose that R2A broth can serve as an appropriate medium for measuring biofilm formation in conditions most relevant to the sink trap and WPS as has been completed previously [99, 100]. However, as mentioned in the Introduction, it is important to bear in mind that disposal of liquids and solids via sink traps will result in variable nutrient conditions and so there is no perfect laboratory model to mimic real-life sink traps or WPS. At both physiological and environmental temperatures in minimal and rich conditions, *C. pauculus* biofilm was evident from 24 h and increased consistently over the 72 h experiments. When studied at 37 °C in R2A broth, the *C. pauculus* MF1 biofilm even overtook that of *P. aeruginosa* PAO1 – a classic model organism for biofilm formation. There has been little previous work published on *C. pauculus* biofilm formation and, to our knowledge, this is the first report using the CV solubilization assay. A study investigating the efficacy of different disinfectants against biofilms containing *C. pauculus* recovered from the International Space Station’s water system found that an 18 h flush of either 6 % H_2_O_2_ solution or a mixture of 3 % H_2_O_2_ and 400 parts per billion colloidal silver effectively reduced the viability of 5 day preformed biofilms to <1 c.f.u. ml^−1^ for up to 3 months. It is noteworthy that in the same study, the control biofilm became predominated with *C. pauculus* at an 8 : 2 ratio with *Burkholderia multivorans *, despite equal seeding of these and two other isolates [101]. The ability of *C. pauculus* to persist in oligotrophic conditions, whilst producing EPS, which may shield it from disinfectants and detergents, reinforces concerns that it may successfully persist in hospital WPS. The ability of *C. pauculus* MF1 to form a biofilm shown in this study, overtaking that of *P. aeruginosa* PAO1 at 37 °C, is concerning and could suggest an ability to establish chronic, recalcitrant infections in humans like *P. aeruginosa* does, particularly in cystic fibrosis patients [65, 102]. It would be valuable to further investigate *C. pauculus* virulence under physiological conditions *in vivo*, perhaps with comparisons between clinical and environmental strains to determine whether potent biofilm formation is a feature of both, or is limited to environmental persistence.

This study cannot identify the source of the *C. pauculus* isolate recovered, thus it is impossible to determine whether the isolate described here originated from a human carrier, or was brought in via another route (e.g. the water supply). Nevertheless, it is concerning that an organism previously described as being of environmental origin, which has recently been implicated in several serious cases of human infection, has been isolated in a known pathogen reservoir in a clinical setting. It is further concerning that the isolate is multidrug resistant, including to the antibiotic of last-resort, colistin, to which it has previously been reported to be susceptible and which has been used therapeutically [17]. While there is clear evidence of *C. pauculus* causing morbidity and mortality, which makes its colonization of a sink trap a problem in its own right, it also poses a potential threat due to the carriage and possible horizontal transfer of ARGs. Further investigation and evidence is needed to be able to determine whether *C. pauculus* has more widely crossed into pathogen reservoirs such as sink traps, or whether the isolate described here was an incidental finding.

## Supplementary Data

Supplementary material 1
