# Supplementary material for: Hospital sink traps as a potential source of the emerging multidrug-resistant pathogen Cupriavidus pauculus: characterization and draft genome sequence of strain MF1
Source: J Med Microbiol. 2022 Feb 3;71(2):001501. doi: 10.1099/jmm.0.001501 (PMC8941954; doi:10.1099/jmm.0.001501)
Supplement: Supplementary material 1 [file jmm-71-1501-s001.pdf]

Supplementary Material

Hospital sink traps as a potential source of the emerging multidrug resistant pathogen *Cupriavidus pauculus*; characterisation and draft genome sequence of strain MF1

R2A broth composition (prepared identically to Oxoid CM0906, omitting agar)

0.5 g L^{-1} yeast extract, 0.5 g L^{-1} proteose peptone, 0.5 g L^{-1} casein hydrolysate, 0.5 g L^{-1} glucose, 0.5 g L^{-1} starch, 0.3 g L^{-1} di-potassium phosphate, 0.024 g L^{-1} magnesium sulphate, 0.3 g L^{-1} sodium pyruvate. Adjusted to pH 7.2 ± 0.2.

Table S1. Characteristics of the draft genome of *Cupriavidus pauculus* MF1 following hybrid assembly and annotation

Variable	Result
Number of contigs	5
Size (bp)	6,758,723
Contigs N50 (bp)	3,676,674
Contigs L50	1
GC content	63.9%
Completeness	99.5%
Contamination	0%
Coarse consistency	98.4%
Fine consistency	95.4%
CDS	6,468
Protein-encoding genes with functional assignment	4,063
Protein-encoding genes without functional assignment	2,405
tRNA	61
Repeat regions	47
rRNA	12

Details of the draft genome given by the PATRIC Comprehensive Genome Analysis tool.

Table S2. Summary statistics of the ONT MinION sequencing run

Variable	Result
Number of reads	77,008
Read length N50 (bp)	15,433
Total bases	580,713,201
Mean read length	7,540.9
Mean read quality	7.9
Median read length	4,039
Median read quality	7.8
Active channels	366

Table S3. Characteristics of the draft genome of the *Cupriavidus pauculus* MF1 isolate using Illumina reads alone

Variable	Result
Number of contigs	66
Size (bp)	6,711,495
Contigs N50 (bp)	382,015
Contigs L50	6
GC content	64.1%
Course consistency	98.4%
Fine consistency	95.4%
CDS	6,483
Protein-encoding genes with functional assignment	4,689
tRNA	55
Repeat regions	0
rRNA	3

Table S4. Top 10 results of Multi-Locus Sequence Typing (MLST) using autoMLST

Reference name	Reference assembly ID	Estimated ANI
Cupriavidus pauculus	GCF_000974605	98.2%
Cupriavidus sp. D384	GCF_001652915	86.9%
Cupriavidus metallidurans NBRC 101272	GCF_001598775	86.7%
Cupriavidus metallidurans	GCF_000709045	86.6%
Cupriavidus metallidurans	GCF_001543455	86.5%
Cupriavidus metallidurans	GCF_000709065	86.4%
Cupriavidus sp. HMR-1	GCF_000319775	86.3%
Ralstonia sp. 25mfcol4.1	GCF_900104095	86.1%
Cupriavidus metallidurans H1130	GCF_000496715	86.1%
Cupriavidus metallidurans	GCF_000709025	85.9%
